# A Context-Aware Accurate Wellness Determination (CAAWD) Model for Elderly People Using Lazy Associative Classification

**DOI:** 10.3390/s19071613

**Published:** 2019-04-03

**Authors:** Farhan Sabir Ujager, Azhar Mahmood

**Affiliations:** Faculty of Computing and Engineering Sciences, Shaheed Zulfikar Ali Bhutto Institute of Science and Technology (SZABIST), Islamabad 44000, Pakistan; farhan@biit.edu.pk

**Keywords:** wellness determination, context awareness, healthcare, smart homes, elderly people, associative classifiers, LAC

## Abstract

Wireless Sensor Network (WSN) based smart homes are proving to be an ideal candidate to provide better healthcare facilities to elderly people in their living areas. Several currently proposed techniques have implementation and usage complexities (such as wearable devices and the charging of these devices) which make these proposed techniques less acceptable for elderly people, while the behavioral analysis based on visual techniques lacks privacy. In this paper, a context-aware accurate wellness determination (CAAWD) model for elderly people is presented, where behavior monitoring information is extracted by using simple sensor nodes attached to household objects and appliances for the analysis of daily, frequent behavior patterns of elderly people in a simple and non-obtrusive manner. A contextual data extraction algorithm (CDEA) is proposed for the generation of contextually comprehensive behavior-training instances for accurate wellness classification. The CDEA presents an activity’s spatial–temporal information along with behavioral contextual correlation aspects (such as the object/appliance of usage and sub-activities of an activity) which are vital for accurate wellness analysis and determination. As a result, the classifier is trained in a more logical manner in the context of behavior parameters which are more relevant for wellness determination. The frequent behavioral patterns are classified using the lazy associative classifier (LAC) for wellness determination. The associative nature of LAC helps to integrate spatial–temporal and related contextual attributes (provided by CDEA) of elderly behavior to generate behavior-focused classification rules. Similarly, LAC provides high accuracy with less training time of the classifier, includes minimum-support behavior patterns, and selects highly accurate classification rules for the classification of a test instance. CAAWD further introduces the ability to contextually validate the authenticity of the already classified instance by taking behavioral contextual information (of the elderly person) from the caregiver. Due to the consideration of spatial–temporal behavior contextual attributes, the use of an efficient classifier, and the ability to contextually validate the classified instances, it has been observed that the CAAWD model out-performs currently proposed techniques in terms of accuracy, precision, and f-measure.

## 1. Introduction

With the advancements in modern healthcare facilities, it has been observed that humans’ life expectancy is on the rise [1]. As a result, there will be increased population of elderly people in the near future. Elderly people are more prone to critical diseases (such as cardiovascular disease, arthritis, osteoporosis, hypertension, and Alzheimer’s disease to name a few) which results in limited physical activity [2]. 

There is a need for constant monitoring of elderly people to provide required daily healthcare and, more importantly, to counter critical emergency healthcare situations. Constant monitoring is an expensive solution in terms of money and time because a dedicated caregiver is required.

**Figure 1:**
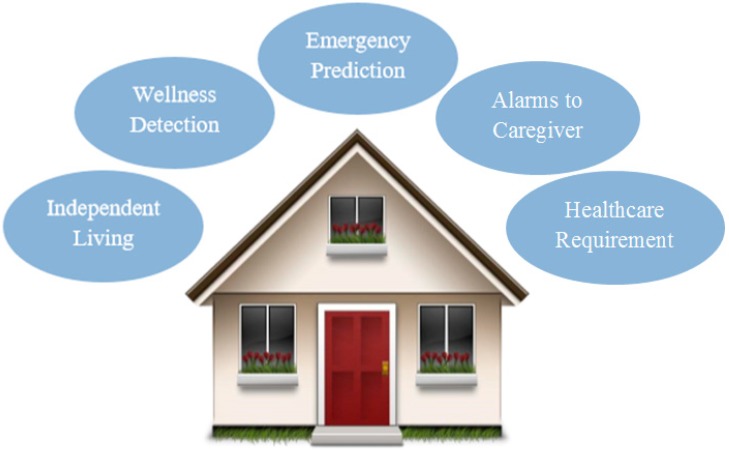
Ambiance for elderly healthcare services.

Today’s modern society is technology driven. More advanced and smarter technologies are emerging to provide optimum solutions to complex problems. There is a requirement for smart, ambient framework which provides constant monitoring of elderly people for independent living (no caregiver), accurate analysis of behavioral patterns for wellness determination, prompt emergency alarms to remote caregivers, and predictions of future healthcare requirements, as depicted in Figure 1.

**Figure 2:**
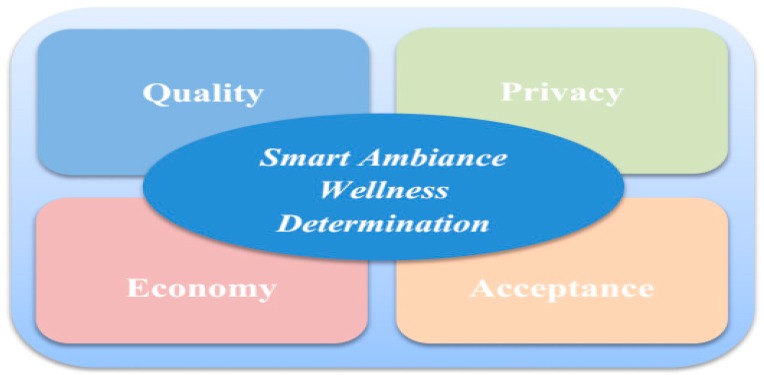
Vital features of smart ambiance elderly healthcare.

An ideal and comprehensive wellness determination framework must have some vital features which ensure the compatibility of the wellness determination mechanism with elderly behavior, such as being easy to use, employing efficient monitoring technology, and having high accuracy in terms of quality. Comprehensive smart ambiance features of wellness determination are portrayed in Figure 2. Elderly people will be more comfortable when their privacy is ensured. Seamless and less complicated monitoring mechanisms will help them to perform their physical activities in a more natural and comfortable manner, thus increasing the acceptance of the smart solutions. These smart solutions should be ideally provided in the living areas of these individuals to ensure the cost-effective and smart solutions are within the reach of every needy individual. Most importantly, these smart-ambiance, wellness-determination solutions should be highly accurate to save precious lives and avoid emergency situations.

Due to these challenges, wellness determination is an active research domain. Several researchers have proposed different wellness determination techniques based on different monitoring technologies. Many researchers proposed wellness determination frameworks on the behavioral monitoring of elderly people, which are based on wearable sensors [3,4]. Wearable sensors-based proposed solutions either monitor vital parameters of the human body or behavioral patterns. Deviation in these features results in determining the wellness of elderly people [5]. Many elderly people are not comfortable wearing sensors constantly; they might forget that they are wearing sensors or forget to charge them, as they are battery operated. As a result, generally elderly people show less acceptability towards wearable sensor-based solutions [6]. Image processing is another research domain which lacks acceptability, as constant visual monitoring lacks privacy [7].

A substantial amount of research work has been done on behavioral monitoring for wellness determination of elderly people [8,9,10]. Numerous wellness classification-based techniques (models and frameworks) have been proposed. These proposed classification models and frameworks are based on artificial intelligence (AI), data mining, and statistical and machine learning-based techniques [5,8,11,12]. Bayesian classifiers have been proposed for wellness determination on behavioral monitoring [8,13]. Similarly, several models have been proposed which are based on the hidden Markov model (HMM) [5,14]. Most of these proposed solutions do not focus on the behavioral monitoring and wellness aspects of individuals, such as the correlation of an activity with its sub-activities, location of an activity, and the time and duration of the activity [15]. Less attention has been given to association mining for wellness determination by researchers, however, association mining is either employed for preprocessing [8,10,11] to analyze frequent behavioral patterns, or for wellness determination classification [12]. Association mining generates global search classification rules on given training data, which are useful in the class prediction [16,17]. However, association mining results in a huge amount of classification rules and, while in pruning phase, many useful rules are pruned. Similarly, fixed support and confidence values for classification rule generation ignores activities with fewer occurrences.

Most of the existing proposed solutions do not consider the temporal and spatial correlation of an activity for better behavioral analysis. Considering these activity parameters will help to translate elderly behavior from the real world to a digital environment for analysis. Association mining provides correlation analysis, but at the cost of a large number of association rules, which results in longer training times of classifiers. Similarly, important correlation rules are ignored in correlation analysis due to low support. In this paper, context-aware accurate wellness determination (CAAWD) is proposed, which aims to provide highly precise and accurate healthcare wellness monitoring, along with the assurance of individual privacy and acceptability (adequacy) consideration to elderly people, which is a weakness in wearable sensors-based and image processing-based wellness solutions. To achieve high classification accuracy and precision, the lazy associative classifier (LAC) technique is employed. The contributions of CAAWD are as follows:

- Spatial–temporal and related contextual parameters correlation of activities of daily living (ADLs) for better behavioral-pattern analysis;
- Introduction of CDEA ensures inclusion of behavioral contextual information for logical training of the classifier for accurate wellness determination;
- Fewer high-quality associative classification rules for efficient classification;
- Rule generation at minimum-support values to ensure that, even with fewer occurrences, training data is not ignored for useful rules;
- Ability to contextually validate the classified instances by introducing caregiver contextual information for elderly people.

The remainder of this paper is arranged as follows: Section 2 discusses literature review on wellness determination in WSN based smart homes and ADL concept and associative classifiers; Section 3 discusses CAAWD; Section 4 presents the experimental setup and results discussion; Section 5 presents the conclusion.

## 2. Related Work

Wellness determination of elderly people in WSN smart homes has recently gained the attention of researchers. Several techniques, models, and frameworks have been proposed from different domains, such as statistical, machine learning, artificial intelligence (AI), and data mining techniques [5,8,11,12]. This section discusses the critical analysis (contributions and limitations) of the existing proposed solutions. 

Wellness of an individual or elderly person can be determined on the basis of the (ADLs) performed [18]. The ADLs concept was introduced by Katz as an aged illness study index. ADLs patterns help one understand the daily routines of elderly people; any deviation from the pre-defined threshold values helps to analyze the wellbeing. The literature review is conceptually divided into ADLs and existing literature of wellness determination of elderly people.

### 2.1. Activities of Daily Living (ADLs)

ADLs are the activities which are performed by people on a daily basis as their routine activities. More importantly, these activities are the activities which are being performed without any assistance. The ADLs index is a concept introduced by Katz [18] to rank the adequacy of an individual to perform ADLs. The ADLs are associated with elderly people and patients. To measure the healthcare and monitoring requirements of an elderly person or a patient, a parameter is introduced known as the ADLs index. This ADLs index helps to determine what type of healthcare and monitoring is required. 

Most of the smart homes projects and labs for elderly healthcare are designed to analyze ADLs for a better understanding of the behavioral patterns, such as Laboratoire d’Intelligence Ambiante pour la Reconnais- sance d’Activite’s (LIARA) Lab [19] and Center for Advanced Studies for Adaptive Systems (CASAS) smart home project [20], to name a few. These labs, with the help of sensors, identify ADLs of elderly people and, via WSN, ADLs data is stored in a central repository for analysis purposes. 

The ADLs consist of some basic routine activities [18], such as eating, bathing, dressing, self-hygiene, walking, and continence. Different analysis models extend ADLs according to certain requirements, for example, watching TV, eating snacks, and outdoor activities to name a few, according to the experimental setup. However, the majority of the literature utilizes the same ADLs for behavior analysis purpose.

**Figure 3:**
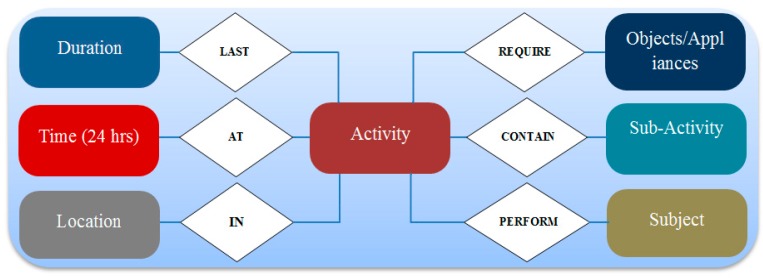
Activities of daily living (ADL) behavioral contextual characteristics for wellness determination.

In [15] a model was proposed on spatial–temporal correlation analysis for the evaluation of complex ADLs. This model highlights the importance of analyzing ADLs on the basis of temporal and spatial correlations of the ADLs, i.e., where and when these ADLs are performed. Temporal and spatial correlations help to analyze the behavioral patterns of elderly people. An ADLs activity is composed of several sub-activities; sequential association and correlation of sub-activities within an activity leads to better behavioral-pattern recognition of an elderly person. Ideally, the features shown in Figure 3 are useful to understand ADLs as behavior-related contextual information of an individual for wellness determination.

### 2.2. Elderly People Wellness Determination and ADLs Detection Classification

In this section, existing proposed models and frameworks are critically reviewed to understand their strengths and limitations.

Supervised probabilistic models are proposed [5,11,12,14,21,22], either for wellness determination or activity discovery of the elderly in smart environments. These proposed solutions are based on the Markovian model and the hidden Markov model (HMM). Markovian and HMM models have the limitation of learning a long range of dependencies in sequence [23]. ADLs are composed of sub-activities and individuals have specific sequential patterns of conducting activities. As a result, Markovian- and HMM-based proposed systems might not give the desired accuracy in analyzing and classifying long, sequential behavioral activities.

Bayesian-based classifiers are employed for wellness determination and activity detection [8,24,25]. In [8], the author analyzed elderly people’s behavior using electric appliance’s power consumption patterns. The behavior of an individual was observed from the power consumption patterns in their living area. Behavioral activities were identified and frequent patterns were discovered regarding the power consumption data. However, electric power consumption patterns do not cover many ADLs, such as toileting, walking, and so on. Similarly, another Bayesian classifier-based solution was proposed in [24], where frequent pattern-mining for behavioral activity analysis was used as preprocessing for the Bayesian classifier. However, Bayesian classifiers have an inability to deal with the attribute dependence [26] and when considering behavioral analysis, ADLs attribute dependence is vital.

Formal concept analysis (FCA) was employed for elderly wellness determination and activity detection in smart ambiance, respectively [27]. Behavioral analysis of an individual was based on the correlation of a specific activity with the sequence of sub-activities in wellness determination and activity detection. However, the concept lattice size can be exponentially large while conducting such analyses, as reported in [28]. Similarly, in concept generation, there is no technical way to be sure that all unique concepts are generated.

An artificial neural network (ANN)-based model was proposed for classification of wellness determination [29]. Frequent pattern-mining was employed to determine the behavioral patterns of an elderly person as preprocessing to an ANN classifier. However, ANN-based classifiers are difficult to retrain, as individual behavior changes with different factors (such as events, weather change, and so on); accommodating such behavior changes would be difficult in such proposed techniques.

In [10], Finite Automata (FA) was proposed to find the disease evolution by monitoring the changing behavior of a patient. Frequent pattern-mining was used to identify frequent behavioral patterns to form Extended Finite Automata (EFA) for activity recognition. However, the conditions of state transitions are fixed. Any slight change in behavior can lead to a false alarm of the wellness of an individual.

Another association rule mining-based ADLs determination-classification model for elderly people was proposed in [16]. This proposed model claimed to determine complex ADLs with high accuracy.

In fact, associative classifiers have not received much attention in WSN-based smart homes wellness determination, due to their tendency to generate a large number of rules. However, associative classifiers have been proved to be potential candidates in other healthcare domains for their high accuracy, as compared to other classifiers [30,31]. Associative classifiers provide high accuracy; during testing phases a global best approach is followed to identify the appropriate classification rule for the test instance, while other classifiers (such as decision trees) follow a greedy approach (local best), as reported in [17]. However, associative classifiers take a large amount of training time, as there is a large number of association rules. Another limitation associated with associative classifiers is that, in the pruning phase, many useful, infrequently occurring rules are pruned [17,32], as they don’t fulfil the minimum support (fixed parameter) criteria.

### 2.3. Associative Classifiers

Data classification consists of two phases, i.e., the training phase, where the classifier is built using training instances and the testing phase, where test instances are classified. Most of the classifiers adopt the eager learning approach, where a classification model is constructed on the provided dataset (training data) and test instances or tuples are classified using the constructed model [32]. Another approach is lazy learning, which exhibits the following three characteristics:

- Deferring the training phase until the request is made, i.e., test instance;
- Classification of the instance is based on the specific training part of the whole dataset;
- Intermediate results and constructed rules projections are discarded.

Due to a global best approach for the identification of classification rules, associative classifiers have shown high accuracy and precision in different domains, such as heart disease prediction [30], image processing classification [31], and complex ADL detection in smart environments [16]. Similarly, in associative classifiers, classification rules are presented in a simple IF–THEN (i.e., IF A THEN B) manner and specific rules can be updated easily without retraining of the whole model—a change in behavior of an elderly person can also be updated easily. These results inspired us to implement associative classifiers for wellness determination of elderly people in smart environments. 

Associative classifiers also work in two phases like other eager learning classifiers. The first phase consists of the training part, where the classifier is trained on the extracted correlated rules based on threshold parameter (support and confidence). A large number of classification rules are extracted by using any association-mining algorithm such as Apriori, FP-growth, or any other algorithm. The training phase is not complete until the pruning of a large set of rules has been done. Various pruning techniques include statistical technique (Chi-square) χ^2^ [33], database coverage [34], pessimistic error estimation [35], lazy pruning [17], conflicting rules, and redundant rules. The next phase is the testing phase, where test instances are classified. 

However, there are a couple of weaknesses in associative classifiers, such as a large number of association rule generations for the training of the classifiers as reported in [17]. Another weakness is the fixed support parameter for the identification of highly correlated rules. Support is not a potential approach [32], as important instances of low support are ignored. Similarly, in the pruning phase many useful rules are pruned, which affects the overall classification accuracy.

#### 2.3.1. Lazy Associative Classifiers (LAC)

LAC was proposed in [17]. It has two-phase training and testing. In the first phase, as an associative classifier, it needs to extract the association rules by using an association mining algorithm. LAC uses Frequent Pattern (FP) Growth association mining algorithm due to its advantages over other similar algorithms. The extracted rules are stored in an FP-tree for efficient retrieval when needed. Rule pruning is not done, as it follows the lazy approach. In the second part, as the test instance arrives at the classifiers for the classification, projections of classification rules are made on the basis of the attributes on test instances from the already derived association rules. Lazy pruning is carried out for these projected rules for minimum pruning of classification rules to avoid the pruning of useful rules. These rules are partitioned into three sets [32]:

Used rules (U_D_): These are the rules from the training data instances which have classified the test instance at least once.
Sparse rules (S_D_): These are the rules which are not used for the classification of any test instance but later turn out to be useful for classification.
Wrong rules (W_D_): These are the rules which have incorrectly classified the training instances. These rules are pruned.

Ranking of the used and sparse rules is done based on the information gain value [32]. Test instance is classified based on the rules having the highest information gain.

## 3. Context-Aware Accurate Wellness Determination Model

The CAAWD aims to provide accurate wellness determination of the elderly living in smart environments. The core feature of the CAAWD is quality, i.e., to have more accurate wellness determination classification of the elderly people. Wellness determination accuracy of the proposed model is improved by converting raw behavior monitoring sensor data into a more meaningful way to ensure the proper training of the model. Ambient information, such as spatial–temporal behavior attributes, is introduced in the CAAWD to define the behavior of an individual in better manner. CAAWD training was done in a comprehensive manner by including low-occurrence behavior routine instances, as these instances are the part of behavior routine. Additionally, classified instances are further validated by the contextual information provided by the caregiver.

### 3.1. Formal Definition of the Concepts in CAAWD Model

The CAAWD is based on the LAC. Formal definitions of the concepts are presented for better understanding of the overall working of CAAWD. CAAWD utilizes the concept of temporal correlation of activities of daily living (ADLs) as proposed in [15]. The definitions of the concepts and correlation rules are as follow:

**Definition** **1.** *The data of interest D is defined by k attributes A_1_, A_2_, …, A_k_, temporal value T and class attribute C, where temporal value T is a set of discretized time value of activities.*

**Definition** **2.** *The training tuple T_RD_ belongs to D, T_RD_ ϵ D, whose class attribute value is known*.

**Definition** **3.** *The testing tuple T_SD_ belongs to D, T_SD_ ϵ D, whose class attribute value is unknown.*

**Definition** **4.** *Association rule is defined as X → Y, where X is antecedent and Y is consequent.*

**Definition** **5.** *Class association rule (CAR) is defined as A → C, where A is a single feature attribute i.e., A ϵ D (A_1_, A_2_, …, A_k_) and T, Where C is a class label.*

**Definition** **6.** *The relative support (ϴ) of the CAR (A → C) is calculated as (X ∪ C) in D.*

**Definition** **7.** *A CAR is a frequent pattern if*

(1) $$ϴ=\frac{\left|X\right|}{\left|D\right|}\geq {ϴ}^{\prime },$$

*where ${ϴ}^{\prime }$ is the minimum support.*

**Definition** **8.** *Entropy H(A) of pattern defined by k values is defined as*

(2) $$H\left(A\right)=\;\sum_{i=1}^{k}P{\left(A\right)}_{i}.\log_{2}\left(P{\left(A\right)}_{i}\right),$$

*where P is representing the probability of an event.*

**Definition** **9.** *Information gain (IG) of a CAR. CAR is represented by A which is a random single feature (attribute) from D i.e., A ϵ D (A_1_, A_2_, …, A_k_)*

(3) $$IG\left(\frac{C}{A}\right)=H\left(C\right)-H\left(\frac{C}{A}\right),$$

*where (C) is representing class feature (attribute), H(C) is the entropy and H(C/A) is conditional entropy.*

**Definition** **10.** *Used rule set (U_D_) is a set of all frequent CARs, which are used at least once for the classification.*

**Definition** **11.** *Sparse rule set (S_D_) is a set of all frequent CARs, which are not used for the classification of any training data instance, but become useful to classify test instance data, which used rules have not.*

**Definition** **12.** *Wrong rule set (W_D_) is a set of all frequent CARs, which have incorrectly classified the training instances.*

**Definition** **13.** *Projected training set (P_TS_) consists of (U_D_), (S_D_), and (W_D_); frequent CARs are ranked on the basis of ascending IG values in these subsets.*

**Definition** **14.** *AB_i_ is an Abnormal classified instance by the data classification module. AB_i_ classification is based on the training of the behavior routine of an individual. Where ACT represents an activity and Sub_ACT represents sub activity.*

AB_i_ = {Start_time, Object, Location, Sub_ACT, ACT, Duration, Class: Abnormal}_i_

**Definition** **15.** *C_j_ is a context defined by the caregiver for a particular elderly person. C_j_ ϵ ACT_j_, ACT_j_ is a predefined activity performed by an elderly person.*

C_j_ = {Frequency_ACT_j__Range, Duration_ACT_j__Range, Start_time_ACT_j__Range}

Frequency_ACT_j__Range defines the range of how frequently an activity is being performed, Duration_ACT_j__Range defines the range of duration (in seconds) of an activity, and Start_time_ACT_j__Range is the range of the start time of an activity. 

**Figure 4:**
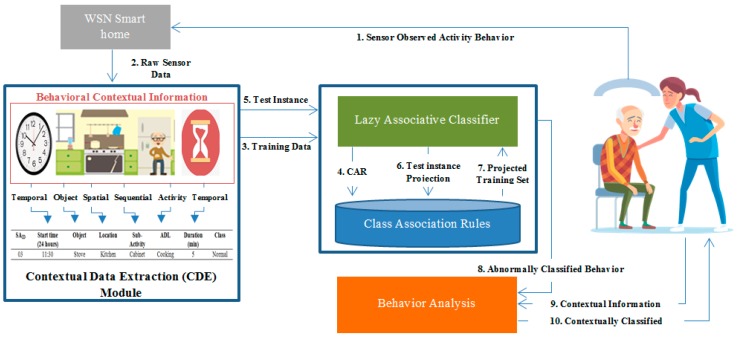
Context aware accurate wellness determination (CAAWD) model for elderly people.

The conceptual design of the CAAWD is depicted in Figure 4. It consists of following modules:

- Contextual data extraction (CDE) module;
- Classification (LAC) module;
- Behavioral analysis (BA) module;
- Caregiver alarm module.

A common smart home environment setup consists [12] of binary sensors installed in household objects and daily-used appliances. These sensors are installed to analyze the behavioral patterns of elderly people. These sensors send ON/OFF states along with main sensor identities (SA_ID_) and temporal values with the help of wireless sensor network (WSN) to the central server for further analysis. These sensor states help to analyze the ADLs of the elderly person living independently in the smart environment.

As discussed earlier in Section 2.1, a behavior pattern of an individual has contextual parameters, such as sub-activities and temporal and location correlation (ambient contextual information) with the behavior pattern. These behavioral contextual parameters have vital significance for the inference of frequent behavioral patterns of an individual. Considering the significance, ambient contextual information is introduced for better learning of the behavior routines of an elderly person. Spatial–temporal contextual information correlation with other contextual parameters (e.g., duration, object used, sub-activities) of the performed activity is of greater importance while learning and classifying an elderly behavior for wellness determination. This is a major contribution of CAAWD, which improves the wellness determination accuracy to a greater extent.

### 3.2. Contextual Data Extraction (CDE) Module 

**Figure 5:**
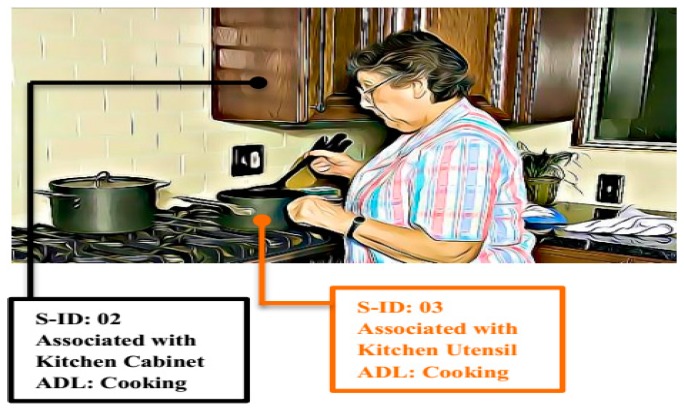
Elderly woman cooking in a smart environment.

Sensor data is transmitted to the server in raw form i.e., 0 s and 1 s, to represent ON and OFF states of the appliances and household objects along the S_ID_. CDE converts these binary values into labeled behavior instances so that correlation mining can be applied at the server. For example, an activity of cooking meal by an elderly woman is taking place in the kitchen, as shown in Figure 5. From her cooking activity, the following information will be extracted by using CDE for context awareness:

- Start time of the activity;
- Object used for the activity;
- Location of the activity;
- Sub-activity;
- Duration of an activity;
- Activity;
- Class (normal/Abnormal).

This main activity will be accomplished by the set of multiple sub-activities, such as utensil selection, cutting or chopping of food, turning on stove, etc. The sequence of these sub-activities in an ADL is important to understand the behavioral pattern of an individual. The start and end times of each sub-activity are represented by a sensor or multiple sensors activation (ON) and deactivation (OFF). The time spent in each state (in case of overlapped activities), is recorded in 24 h time format.

The location of an ADL is identified from the SA_ID_. Analysis server has mapping of all the sensors according to the installation location; a specific SA_ID_ represents a specific location and ADL. Sub-activities are also identified with the help of sub-activity sensor ID (SS_ID_) at the server. Sub-activities will only be recognized at the server if SA_ID_ is active (ON), thus behavior patterns (ADLs) of an elderly person can be uniquely analyzed with respect to sub-activities at the server. The duration attribute is only considered for the main activity (ADL) not for the sub-activity. For CAAWD, basic ADLs are considered, which are discussed in Section 4. 

Contextual data was extracted and presented in Table 1 as dataset instances for the discussed example of Figure 5.

A contextual data extraction algorithm (CDEA) is proposed for generating contextually comprehensive training instances for better training of classifiers and hence more accurate wellness classification.

**Algorithm 1** Contextual data extraction algorithm (CDEA)Input: Sensor identities (SA_ID_, SS_ID_, ACT_ST_, ACT_ET_), where SA_ID_ is the main activity, SS_ID_ is the sub-activity identities, ACT_ST_ and ACT_ET_ are the activation and deactivation time of SA_ID_ (representing a main activity) respectively.Output: Comprehensive training tuple (Time ^ Location ^ ACT ^ S_ACT ^ Dur_ACT → Class)1: IF (SA_ID_== ON state)2:  IF (SA_ID_ == i)3:     Location = Loc_i_ ^ Activity = ACT_i_ ^ Object = Obj_i_4:  IF (SS_ID_ == j)5:     Sub_Act = S_ACT_j_6: IF (SA_ID_ == OFF state)7:        Dur_ACT_i_ = |ACT_ET_ − ACT_ST_|8:    ELSE  Dur_ACT_i_ = undetermined9:  Time = ACT_ST_10: RETURN Training tuple (T_RD_)

CDEA has prior knowledge of all the sensors identities and their locations (spatial contextual information) of installation. When a sensor senses an activity, it transmits the ON state to the sink along its SA_ID_. The CDEA at the sink identifies the main activity based on SA_ID_ (as sensors are installed in specific household objects or appliances, which are representing specific activities) and the activity location. Behavior routine patterns of an elderly person are signified with the help of contextual information (location and object of usage) of an activity for better training of the classifier. As discussed earlier, it is important to correlate sub-activities with main activities for better training of the classifier. For this purpose, sensors representing main activities (SA_ID_) are differentiated from sensors representing sub-activities (SS_ID_). The duration of a main activity is calculated from the activation time (ACT_ST_) until the deactivation time (ACT_ET_) of the SA_ID_. The duration of an activity will remain “undetermined” until deactivation of the SA_ID_ is observed. Finally, temporal contextual information (activity start time) is introduced in the training instance. CDEA will return a comprehensive training instance (T_RD_) that defines an activity wellness pattern in a logical manner. CDEA generates comprehensive training instances, as these instances are representing the contextual behavior patterns with respect to spatial information (location), temporal information (time), duration of an activity, object used for an activity, and correlation of main activity with sub-activity.

CDEA is proposed, keeping in mind the working mechanism of associative classifiers. The classifier will generate frequent patterns in a more understandable manner, which will not only be used for wellness determination but can also be utilized in behavioral analysis by an expert or caregiver. Similarly, the introduction of behavioral contextual information by CDEA is ideal while considering LAC projection-based classification, where vital contextual information is introduced as wellness parameters/attributes. When a test instance arrives for classification with any abnormal attributes (e.g., abnormal activity time, longer or shorter duration of an activity, out of sequence activities or any other), classification rules will be projected according to behavior attributes and the classifier will determine the wellness based on these contextually defined rules. 

### 3.3. Classification Module 

This section explains the wellness determination classification process. For CAAWD, LAC is preferred as it gives better accuracy and precision than its other counterparts as reported in [23,31], but it has never been proposed and evaluated for wellness determination of elderly people living in smart environments. To the best of our knowledge, it was proposed and evaluated for the first time for the wellness determination of elderly people in WSN smart homes. This section also discusses the relation of information gain and low-support value for better CAR generation for the training of LAC and class prediction.

Association mining was performed on the preprocessed and prepared comprehensive training dataset D based on defined threshold *ϴ*′. As a result of association mining, a CARs dataset was generated and stored as shown in Figure 4 (step 4). CARs were the set of frequent behavior patterns of an elderly person, for example, an elderly person’s frequent sleeping pattern concerns them going to sleep at around 22:30 h. This pattern would be considered as a CAR when a test instance arrives to determine whether it is a normal or abnormal test pattern. The CAR dataset is partitioned in to U_D_, W_D_, and S_D_, as discussed in Section 2.3.1. Table 2 represents sample CARs to understand the significance of CDEA.

As the T_SD_ test instance arrives at the classifier, the classifier requests (based on the attributes of T_SD_) P_TS_ to the database D where CARs are stored, as shown in Figure 4 (step 6 and 7). On reply of P_TS_, P_TS_ contains U_D_, i.e., used CARs dataset, S_D_, i.e., sparse CARs dataset, and W_D_, i.e., wrong CARs dataset. W_D_ is pruned based on the lazy pruning techniques. An important point to consider is that projected CARs will have those rules which have test instance attributes, thus a more concise set of rules. CARs in U_D_ and S_D_ are ranked on the basis of information gain (IG). The test instance will be classified on the basis of the highest IG of a CAR from the U_D_, or if required from the S_D_. LAC pseudocode is presented as:

**Algorithm 2** LACInput: Set of Rule Set of CARs (D) and set of test instance (T_SD_)Output: Classified instance  1: D (set of RuleSet of CARs)  2:  T_SD_ (set of Test instance)  3:  t_i_ ϵ T_SD_  4:  Let P_TS_ be the projection of D based on the features of t_i_  5:  (U_D_||S_D_) ϵ P_TS_//, where U_D_ is the set of used rules and S_D_ is the set of sparse rules  6:  Sort CARs based on the information gain of P_TS_  7:  Classify the t_i_ based on the highest information gain from P_TS_

To achieve better accuracy and precision of the classifier, CARs ranking is based on IG. Mathematical proof is presented to understand the relationship of IG with ϴ or a mined CAR from [36].

Let a pattern, i.e., CAR, be defined by a single feature A. The IG of this CAR defined by A is defined in Equation (3) in Section 4.1. 

We need to limit the IG and H(C/A) functions by defining the upper and lower bounds. Let IG (C/A)_UP_ be the upper boundary of information gain of the pattern and H(C/A)_LOW_ be the lower boundary of conditional entropy.

(4) $$IG{\left(\frac{C}{A}\right)}_{up}=H\left(C\right)-H_{low}\left(\frac{C}{A}\right)$$

The support of pattern CAR (by a random feature A) is defined by ϴ. H(C/A)_LOW_ is the conditional probability, which can be defined with the help of Definition 10. For simplification, certain assumptions are considered. These assumptions are: A ϵ {0,1} and C is the class attribute which is binary, so C ϵ {0,1}. Let P(c = 1/a = 1) = q, P(c = 1) = p, and P(a = 1) = ϴ. Where p and q are variables, then

(5) $$\left(C/A\right)=-\sum_{a\in \left\{0,1\right\}}P\left(a\right)\;.\sum_{c\in \left\{0,1\right\}}P\left(\frac{c}{a}\right)\log P\left(\frac{c}{a}\right),$$

(6) $$H\left(C/A\right)=-ϴ\;q\;log\;\left(q\right)-ϴ\left(1-q\right)\log\left(1-q\right)+\left(ϴq-p\right)\log\frac{p-ϴq}{1-ϴ}+\left(ϴ\left(1-q\right)-\left(1-p\right)\right)\log\frac{\left(1-p\right)-\;ϴ\left(1-q\right)}{1-ϴ}.$$

Taking the partial derivative H(C/A)_LOW_ with respect to ϴ,

(7) $$\begin{array}{c} \frac{\partial H{\left(\frac{C}{A}\right)}_{q=1}}{\partial ϴ}=\log\frac{p-ϴ}{1-ϴ}-\frac{p-1}{1-ϴ}-\frac{1-p}{1-ϴ}\\ \;=\log\frac{p-1}{1-ϴ}\\ \;\;\leq \log1\\ \;\;\;\;\leq 0. \end{array}$$

This mathematical analysis discusses the relationship of information gain (IG) with the support (ϴ). The larger the value of H(C/A)_LOW_, the smaller the value of ϴ and (IG). Thus, the low-frequency patterns are bound by small value of (IG) and ϴ. The minimum value of ϴ ensures the inclusion of low-frequency patterns for greater importance for the higher accuracy and precision of the classification.

CDEA’s comprehensive training instances (CARs) ensure the inclusion of vital contextual information for wellness determination by the classifier. Projection based classification of LAC along with CDEA helps CAAWD to achieve higher wellness accuracy. Wellness determination based on the contextual behavior information is highlighted in CAAWD for better accuracy. 

### 3.4. Behavior Analysis (BA) Module 

Test instances classified as “normal” are provided to the caregiver; abnormal-classified (AB_i_) cases will be provided as input to the “behavior analysis” module, according to Definition 14. This module analyzes AB_i_ based on the contextual information C_j_ provided by the caregiver, according to Definition 15. Based on the analysis, abnormal-classified instances will finally be observed as normal or abnormal. The classifier can classify a given test instance as abnormal but contextually it can be normal, or vice versa. For example, a CAR based on the behavior routine and a test instance is shown in Table 3 and Table 4. 

According to the classifier generated CAR, this test instance would be considered as an abnormal activity, according to the provided training data. The contextual information in the behavior analysis module would help to correct the test instance from abnormal to normal, as this contextual information is not part of the regular behavior routine, but recommended by the caregiver/expert. The caregiver defines the context according to Definition 15 for each activity. The proposed algorithm (context awareness (CA)) is presented below.

**Algorithm 3** Context Awareness (CA)**Input:** Abnormal classified instance (**AB_i_**) and context (**C_j_**)**Output:** Context aware classified instance (**CACI**) 
1:AB_i_ = {Start_time_i_, Object_i_, Location_i_, Sub_ACT_i_, ACT_i_, Duration_i_, Class: Abnormal}//Definition 14

2: C_j_ = {Frequency_ACT_j__Range, Duration_ACT_j__Range, Start_time_ACT_j__Range}// Definition 15

3:IF (ACT == ACT_i_)

4:   Frequency_ACT_i_ = Frequency_ACT_i_ ++

5:IF (Duration_i_ϵDuration_ACT_j__Range AND Start_time_i_ϵStart_time_ACT_j__Range AND Frequency_ACT_i_ϵFrequency_ACT_j__Range)

6:             Class = Normal

7:        ELSE Class = Abnormal


The abnormally classified instance is analyzed by the proposed algorithm (CA). According to Definition 15, behavioral context is provided by the caregiver with respect to frequency, start time, and duration of an activity. CA will check the abnormally classified instance with respect to these three defined pieces of contextual information (i.e., frequency, duration and start time of main activity) using (AND) logic. If an abnormally classified instance violates any contextual information it will remain as abnormal, otherwise it will be made normal. This is another major contribution of the CAAWD, as all classification models use training data for classification, however, in such application areas (such as healthcare and wellness determination) requirements change with the passage of time and training data is not sufficient for accurate wellness determination. 

### 3.5. Caregiver Alarm Module 

A remote caregiver will be continuously monitoring the wellness of an elderly person and, in case of abnormal classified activity, an alarm will be generated and the caregiver will be notified of the description of the abnormal behavior, such as long sleep durations, the usual behavioral pattern, the stationary position of an individual, and any activities being performed in abnormal locations (for example, sleeping in the living room). Temporal attributes of an observed behavior pattern helps in the identification of abnormal behavior. If an elderly person is in the habit of taking a nap on the sofa (living room) in the afternoon (15:30 to 16:45), this behavior pattern will be observed in the frequent behavior patterns and will be treated as a CAR. Supposing the same elderly person is sleeping on the sofa (living room) at 23:40 to 4:30, this pattern will deviate temporally from the frequent pattern and result in the abnormality. Such information will be sent to the remote caregiver for a better understanding of the elderly person’s health situation; this information will be significant for the caregiver to suggest the type of medical assistance and services required before any critical emergency situation can occur. CAAWD analyzes the frequent behavior routine patterns of an elderly person and determines the wellness.

## 4. Experimentation

In this section of dataset descriptions, the evaluation of the CAAWD and comparison of the CAAWD with the exiting proposed models and techniques has been conducted.

### 4.1. Ordonez’s ADL Dataset

This dataset [12] consists of ten individuals performing ADLs in a similar four room smart home environment for fourteen days. These smart environments are equipped with binary sensors for the behavioral monitoring of the individual. The description of ADLs and sensors for the two subjects is given in Table 5. 

### 4.2. Evaluation Metric 

This subsection explains the selected parameters for the evaluation of the CAAWD. As discussed earlier, the efficacy of the CAAWD is based on how accurate the wellness of the elderly can be determined. Wellness determination has been evaluated for accuracy, precision, and f-measure metrics. The CAAWD handles the binary classification of normal or abnormal behavior of the elderly person. Training instances are balanced by using class balancing preprocessing techniques. 

Accuracy explains the ratio of correctly predicted instances over the total number of the instances that are evaluated by the classifier. Mathematically, accuracy is explained in Equation (8).

(8) $$Accuracy=\frac{TP+TN}{TP+TN+FP+FN}$$

The f-measure represents the weighted average of the precision and recall metrics. Mathematically, the f-measure is explained in Equation (9).

(9) $$F-Measure=2\times \frac{Precision\;\times \;Recall}{Precision+Recall}$$

Precision and recall are mathematically defined in Equations (10) and (11), respectively.

(10) $$Precision=\frac{TP\;}{TP+FP},$$

(11) $$Recall=\frac{TP}{TP+FN}.$$

### 4.3. Experimental Setup 

This section describes the experimental setup adopted in the current article. In this research article, the dataset is utilized for the experimentation, as discussed earlier. The proposed algorithm CDEA, LAC, and CA have been implemented in JAVA programming language over the computer system, which serves as the analysis server (CAAWD). Microsoft Structured Query Language (SQL) database is used for storing the original unprocessed dataset and training dataset D, including CARs. Dataset instances are provided to the CDEA to produce contextually comprehensive training instances; these training instances are stored in the database (D). LAC utilizes these training instances for the generation of CARs, as discussed earlier. Similarly, contextual information provided by the caregiver is also stored in the database; 10-fold cross validation has been adopted for the experiment evaluation of the CAAWD. The results are discussed in a later section. 

### 4.4. Results and Analysis

The CAAWD has been evaluated on accuracy, precision, recall, and f-measure metric against existing proposed techniques [5,37]. The existing proposed techniques [5,37] have utilized datasets [12], however [5] has been evaluated on accuracy, while [37] has been evaluated on f-measure.

**Figure 6:**
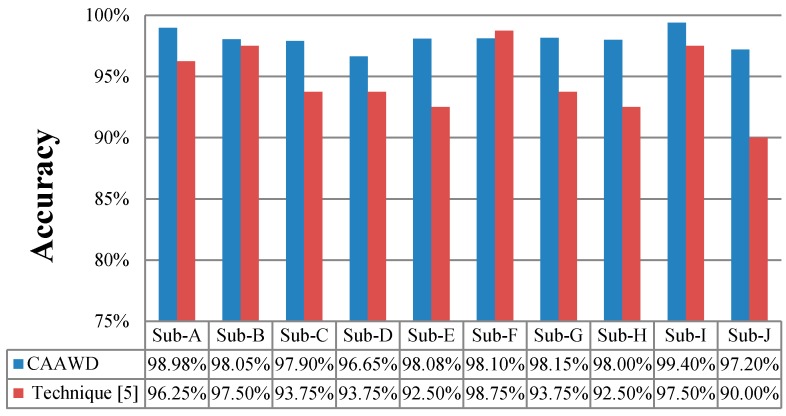
Wellness accuracy of CAAWD against the proposed technique [5] on Ordonez’s dataset.

In Figure 6, wellness determination accuracies of ten individuals/subjects (Ordonez’s dataset) have been determined by utilizing CAAWD and compared against the existing proposed technique [5]. It has been observed that CAAWD is performing better in nine out of ten subjects (i.e., individuals) referred as Sub in Figure 6, due to its associative approach in classification rules generation and global search for the classification. Introduction of ambient contextual information (time and location) and activity contextual information (duration, object, and sub-activity) improves the wellness determination learning ability of the classifier in a more logical manner. CDEA and LAC enhance the accuracies over the power of association mining of an activity’s contextual information. The comprehensive training set preparation by the CDEA improves overall wellness determination over the inclusion of low-support training instances and the introduction of strong associated activity parameters-based CARs. 

**Figure 7:**
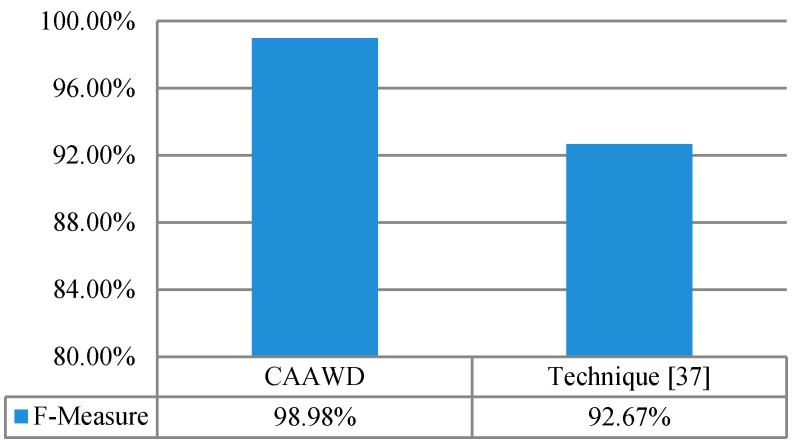
F-measure of CAAWD against the proposed technique [37] on Ordonez’s dataset.

**Figure 8:**
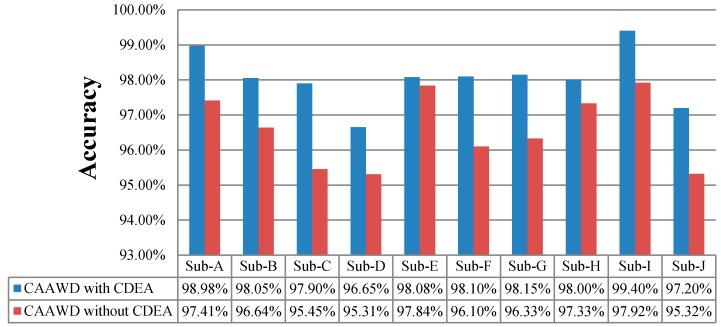
Wellness accuracies of CAAWD with and without utilizing CDEA.

In Figure 7, the wellness determination is evaluated in terms of f-measure. For performance evaluation of CAAWD, the f-measure was determined and compared against the f-measure value observed in [37]. In [37], only one individual-A (Ordonez dataset) f-measure was determined. F-measure considers both precision and recall evaluation metrics. CAAWD performance has been evaluated with and without CDEA in Figure 8. 

CAAWD determines strong associations between spatial–temporal and other activity contextual parameters by using CDEA. These associations help in the generation of better classification rules. Inclusion of contextual information (by CDEA) ideally fits in with the working mechanism of LAC. The lazy approach uses three sets of rules, as discussed in Section 3.1; with the large number of association classification rules, projection of test instance attributes yield more accurate and highly correlated wellness determination classification rules. As a result, we can observe better accuracy while CAAWD utilizes CDEA. 

In Table 6, classification parameters (precision, recall, and f-measure) are presented for each individual.

For the context aware validation, we have generated 2000 behavior anomalies for each elderly person and experimented with CAAWD for the proposed behavior analysis (BA) module validation. Of these 2000 anomalies, 1000 are “normal” and 1000 are “abnormal”. Out of 1000 “abnormal” activities, 500 are contextually “normal” and 500 are contextually “abnormal”. Table 7 represents the possible behavior anomaly examples and possible reasons.

The LAC classifier classifies these generated instances for ten subjects as normal/abnormal and is represented as in Table 8.

For validation of the behavioral analysis (BA) module, only abnormally classified instances are considered and the following contextual information has been utilized, as shown in Table 9.

The following results of ten subjects have been observed. Their accuracy, precision, recall, and f-measure values were calculated and are presented in Table 10. Table 11 presents the comparison of the weighted (accuracy, precision, recall, and f-measure) values of CAAWD and the existing proposed technique [5]. 

It has been observed that the BA module has successfully introduced contextual information and, with the analysis of each abnormal instance, it has classified with higher accuracy according to the provided contextual information. It has also been observed that accuracy has fallen due to the violation of contextual conditions provided for validation. The weighted accuracy and precision of the technique proposed in [5] is observed as 95.1% and 94.9%. We have observed promising results, as CAAWD has successfully outperformed the weighted accuracy and precision of the technique proposed in [5], as presented in Table 10.

One of the inherited limitations of the associative classifiers is that associative classifiers take longer to build a classification model. LAC has been analyzed with respect to the time taken to build the classification model (seconds) in Table 11. These experiments have been conducted on a core i5 processor and a 4 GB RAM computer system on Ordonez’s dataset. The average time to build a model (seconds) is presented in Table 12. 

It is observed that LAC has a shorter classification model building time than WAC (associative classifier), but is longer than Naïve Bayesian and C 4.5, due to its lazy classification approach. 

### 4.5. Discussion

It has been observed that CAAWD performs better in accuracy, precision, recall, and f-measure by utilizing dataset [12], when compared against the existing proposed techniques. However, CAAWD needs to be analyzed in a real smart home environment. CAAWD is based on location specific activity; this limitation can be eliminated by utilizing wearable sensors which will help in identifying the other ADLs, however, such datasets should be provided for further investigation. LAC’s model training time is better than other associative classifiers because of its lazy classification approach, however, other classification models, such as C 4.5 and Naïve Bayesian, have better training time than LAC. CAAWD determines the wellness of an elderly person using behavior pattern analysis; CAAWD needs to be further investigated in several contextual scenarios, such as weather change, web usage, and the medical vital parameters of an elderly person. Similarly, outdoor monitoring of elderly people needs to be integrated for a comprehensive wellness determination. 

## 5. Conclusions

In this research article, a model for accurate wellness determination of elderly people living alone in smart homes was proposed. The CAAWD aims to provide healthcare monitoring of elderly people, along with the prime object of higher wellness determination classification accuracy and precision for better healthcare. Activities of daily living (ADLs) are monitored via WSN to understand the normal and abnormal behavioral patterns of an individual living in a smart home environment. The contextual data extraction (CDE) module was introduced in CAAWD to highlight the significance of a behavior’s spatial–temporal information (time and location), along with activity associated contextual information (duration, object, and sub-activity) for wellness determination. CDEA generates comprehensive behavior training instances for better training of the classifier; wellness determination is based on significant wellness contextual parameters (time, location, duration, object, and sub-activity). CAAWD employs the lazy associative classifier (LAC) for the first time for the wellness determination of elderly people. The association mining process in LAC generates highly correlated class association rules (CARs). The lazy approach ensures less pruning of useful CARs and the introduction of minimum support behavior patterns. Based on normal and abnormal behavioral patterns, wellness is determined by CARs. CAAWD’s behavior analysis module further analyzes the abnormally classified instances of the contextual information provided by the caregiver, as classification models are trained on the limited provided information and the behavior of individuals changes with time and healthcare requirements. Upon abnormal classification of a behavioral pattern, an alarm is generated to the remote caregiver to avoid emergency situations. CAAWD is evaluated over a benchmark dataset and compared with existing proposed techniques. It has been observed that CAAWD outperformed the compared techniques over the dataset in terms of accuracy, precision, and f-measure. 

The current study could be extended to accommodate the changing behavioral patterns of elderly people with extrinsic contextual information, such as caregiver input, emotions, and important events, as future directions for the existing model.

## Figures and Tables

**Table 1:** Data instances representation of single sensor value by Contextual Data Extraction Module.

SA_ID_	Start Time (24 h)	Object	Location	Sub Activity	ADL	Duration (min)	Class
03	11:30	Stove	Kitchen	Cabinet	Cook	-	Normal
03	11:30	Stove	Kitchen	Cabinet	Cook	-	Normal
03	11:31	Stove	Kitchen	Cooktop	Cook	-	Normal
-	-	-	-	-	-	-	-
03	11:35	Stove	Kitchen	-	Cook	5	Normal

**Table 2:** Generated class association rules (CARs).

SA_ID_	Start Time (24 h)	Object	Location	Sub Activity	Duration (min)	ADL	Class
06	16:30	Sofa Chair	Living Room	-	45	TV	Normal
06	2:30	Shower	Toilet	-	200	-	Abnormal
10	7:33	Shower	Toilet	Wash Basin	15	Shower	Normal
01	6:15	Door	-	-	35	Walk	Normal

**Table 3:** Class Association Rule.

SA_ID_	Start Time (24 h)	Object	Location	Sub Activity	Duration (min)	ADL	Class
13	23:30	Bed	Bedroom	-	7:45	Sleeping	Normal

**Table 4:** Test instance.

SA_ID_	Start Time (24 h)	Object	Location	Sub Activity	Duration (min)	ADL	Class
13	9:30	Bed	Bedroom	-	11:30	Sleeping	?

Contextual information: Bed rest recommended = 12 h.

**Table 5:** ADLs with associated sensors in Ordonez’s dataset.

Serial Number	ADLs	Installed Sensors
1.	Leaving	Magnetic (main door)
2.	Toileting	Passive Infrared (PIR) (basin), flush (toilet)
3.	Showering	PIR (showering)
4.	Sleeping	Pressure (bed)
5.	Breakfast	PIR (cooktop, microwave), electric (toaster), magnetic (fridge, cabinet, cupboard)
6.	Lunch	PIR (cooktop, microwave), magnetic (fridge, cabinet, cupboard)
7.	Dinner	PIR (cooktop, microwave), magnetic (fridge, cabinet, cupboard)
8.	Snack	Electric (microwave, toaster), magnetic (fridge)
9.	Spare time/TV	Pressure (seat)
10.	Grooming	Magnetic (cabinet)

**Table 6:** Classification parameters of each individual.

Individuals	Precision	Recall	F-Measure
Subject-A	98.04%	99.80%	98.91%
Subject-B	98.23%	97.85%	98.04%
Subject-C	97.66%	98.23%	97.94%
Subject-D	95.79%	97.47%	96.62%
Subject-E	97.85%	98.23%	98.04%
Subject-F	97.85%	98.43%	98.14%
Subject-G	97.66%	98.43%	98.04%
Subject-H	97.28%	98.81%	98.04%
Subject-I	99.40%	99.40%	99.40%
Subject-J	96.90%	97.66%	97.28%

**Table 7:** Behavior anomaly examples.

Behavior Routine Anomalies	Possible Reasons
Sleeping longer than usual	Medication, fatigue, hypertension, dizziness
Higher frequency of toileting	Stomach upset, higher diabetes level
Zero duration of leaving	Feeling unwell, skipping daily walk, fatigue

**Table 8:** Classified instances accuracies.

Subject	Normally Classified	Abnormally Classified
Subject-A	98.6 %	97.6 %
Subject-B	97.9 %	98.5 %
Subject-C	99.3 %	97.4 %
Subject-D	99.1%	97.9 %
Subject-E	98.3 %	97.4 %
Subject-F	97.1 %	97.4 %
Subject-G	98.6 %	99.3 %
Subject-H	97.7 %	98.6 %
Subject-I	99.2 %	97.5 %
Subject-J	98.9 %	97.7 %

**Table 9:** Information provided by the caregiver.

Activity	Contextual Information					
	Frequency		Duration		Start Time	
	Min	Max	Min	Max	Min	Max
Sleeping	2	3	8 h	12 h	22:30	23:30
Leaving (walk/exercise)	1	2	30 min	60 min	10:30	15:30
Toileting	4	8	1 min	10 min	-	-
Breakfast	1	1	15 min	35 min	9:30	10:30
Lunch	1	1	25 min	50 min	13:00	15:30
Dinner	1	1	25 min	50 min	18:30	21:30
Showering	1	3	10 min	15 min	-	-

**Table 10:** Subject (individual) wise accuracy, precision, recall, and F-measure representation.

Subject	Accuracy	Precision	Recall	F-Measure
Subject-A	99.11%	98.81%	99.40%	99.11%
Subject-B	98.23%	98.43%	98.04%	98.23%
Subject-C	97.94%	97.66%	98.23%	97.94%
Subject-D	98.14%	98.04%	98.23%	98.14%
Subject-E	97.28%	97.09%	97.47%	97.28%
Subject-F	98.52%	98.81%	98.23%	98.52%
Subject-G	97.37%	98.04%	96.71%	97.37%
Subject-H	99.11%	99.01%	99.21%	99.11%
Subject-I	96.81%	97.09%	96.53%	96.81%
Subject-J	98.33%	98.62%	98.04%	98.33%

**Table 11:** Accuracy, precision, recall, and f-measure comparison of the CAAWD with the technique in [5].

Techniques	Weighted Accuracy	Weighted Precision	Weighted Recall	Weighted F-Measure
CAAWD	98.08%	98.16%	98.01%	98.08%
Technique [5]	95.10%	94.90%	95.10%	94.60%

**Table 12:** Average time to build a classification model using different classifiers.

Classification Model	Average Time to Build Classification Model (s)
Naïve Bayesian	0.05
Decision Tree based Classification (C 4.5)	0.3
Lazy Associative Classifier (LAC)	0.37
Weighted Associative Classifier (WAC) *	41

* Minimum support and confidence values are 0.1 and 0.9 respectively with maximum 10 rules.
